# Preferences for Prenatal Tests for Cystic Fibrosis: A Discrete Choice Experiment to Compare the Views of Adult Patients, Carriers of Cystic Fibrosis and Health Professionals

**DOI:** 10.3390/jcm3010176

**Published:** 2014-02-14

**Authors:** Melissa Hill, Ranjan Suri, Edward F. Nash, Stephen Morris, Lyn S. Chitty

**Affiliations:** 1 Clinical and Molecular Genetics, Institute of Child Health and Great Ormond Street Hospital for Children NHS Foundation Trust, London WC1N 3BH, UK; E-Mail: l.chitty@ucl.ac.uk; 2 Department of Respiratory Paediatrics, Great Ormond Street Hospital for Children NHS Foundation Trust, London WC1N 3JH, UK; E-Mail: ranjan.suri@gosh.nhs.uk; 3 West Midlands Adult Cystic Fibrosis Centre, Heart of England NHS Foundation Trust, Birmingham B9 5SS, UK; E-Mail: edward.nash@heartofengland.nhs.uk; 4 Research Department of Applied Health Research, University College London, London WC1H 0AJ, UK; E-Mail: steve.morris@ucl.ac.uk; 5 Fetal Medicine Unit, University College London Hospitals NHS Foundation Trust, London WC1E 6DB, UK

**Keywords:** non-invasive prenatal diagnosis (NIPD), discrete choice experiment, cystic fibrosis

## Abstract

As new technologies enable the development of non-invasive prenatal diagnosis (NIPD) for cystic fibrosis (CF), research examining stakeholder views is essential for the preparation of implementation strategies. Here, we compare the views of potential service users with those of health professionals who provide counselling for prenatal tests. A questionnaire incorporating a discrete choice experiment examined preferences for key attributes of NIPD and explored views on NIPD for CF. Adult patients (*n* = 92) and carriers of CF (*n* = 50) were recruited from one children’s and one adult NHS specialist CF centre. Health professionals (*n* = 70) were recruited via an e-mail invitation to relevant professional bodies. The key attribute affecting service user testing preferences was no miscarriage risk, while for health professionals, accuracy and early testing were important. The uptake of NIPD by service users was predicted to be high and includes couples that would currently decline invasive testing. Many service users (47%) and health professionals (55.2%) thought the availability of NIPD for CF would increase the pressure to undergo prenatal testing. Most service users (68.5%) thought NIPD for CF should be offered to all pregnant women, whereas more health professionals (68.2%) thought NIPD should be reserved for known carrier couples. The implications for clinical practice are discussed.

## 1. Introduction

Cystic fibrosis (CF) is an autosomal recessive disorder caused by mutations in the CFTR gene. The prevalence is one in 2500 to one in 3500 live births, with a carrier frequency of one in 25 for those with Northern European ancestry [1]. While CF impacts many body systems, the lungs and digestive system are particularly affected. People with CF suffer frequent chest infections, resulting in progressive lung function decline and ultimate respiratory failure. Multi-disciplinary care is vital, focussing on slowing lung function decline, the maintenance of adequate nutrition and psychosocial support, with lung transplantation as a potential ultimate treatment option [2]. Outcomes for people with CF continue to improve, and median survival, which is currently 43 years in the United Kingdom (UK), has significantly improved as a result [3,4].

Testing for CF in pregnancy can be used to plan and prepare for the birth of an affected child or for the option of terminating the pregnancy. People who are carriers of CF and those affected with CF value the availability of prenatal diagnosis for couples with a high genetic risk of having a child with CF [5]. However, prenatal testing for CF is currently reliant on invasive diagnostic tests (chorionic villus sampling or amniocentesis), which have a risk of miscarriage of around 0.5% to 1% [6], and is not performed before 11 weeks in pregnancy in the UK. In the near future, prenatal testing for CF could be performed using non-invasive prenatal diagnosis (NIPD) based on the analysis of cell-free DNA (cfDNA) in maternal plasma. NIPD only requires a maternal blood sample and, thus, avoids the risk of miscarriage associated with invasive diagnostic tests and can be performed early in pregnancy (7–9 weeks). NIPD has been shown to be successful for the diagnosis of CF in cases where parents carry different CF mutations, where it has been used to determine the presence or absence of the paternal mutation [7,8,9]. When parents carry the same mutation, NIPD is more difficult, due to the high background of maternally-derived mutant alleles. New technologies, such as digital PCR and next generation sequencing (NGS), are bringing the possibility of using NIPD for CF closer to clinical implementation by allowing the quantification of normal and mutation alleles (for a review, see [10]).

Research examining stakeholder views is critical for the development of implementation strategies for NIPD for CF and other single gene disorders. Discrete choice experiments (DCE) are a valuable tool for exploring stakeholder preferences and understanding the drivers of healthcare decisions [11]. A DCE provides an opportunity to explore real-life decision making by presenting a series of hypothetical healthcare options with differing attributes and asking participants to indicate which one they would choose, thus enabling insight into people’s preferences and the importance they place on particular attributes [12]. For example, DCEs have been used to examine preferences for screening and diagnostic tests for Down syndrome [13,14,15,16,17,18]. Our previous research has used qualitative approaches to explore the opinions of service users [19] and health professionals [20] around NIPD for sickle cell disease, thalassaemia and CF. The current study utilises a larger scale quantitative approach that incorporates a DCE to compare the views of patients with CF and carriers of CF with those of health professionals who provide counselling for prenatal testing for CF (genetic counsellors and clinical geneticists). Here, we explore the preferences for the key attributes of diagnostic tests for CF, examine views on NIPD, including expected uptake and how testing should be offered, and assess differences in preferences between stakeholders, to inform strategies for appropriate implementation.

## 2. Methods

### 2.1. Ethical Approval

Ethics approval was obtained from the National Research Ethics Service Committee (10/H0714/3). The study design and analysis followed DCE guidelines [12,21,22].

### 2.2. Recruitment of Participants and Data Collection

Data was collected from three groups of participants: (1) adult patients with CF; (2) carriers of CF; and (3) health professionals who offer prenatal testing for CF (genetic counsellors and clinical geneticists). Adult patients and carriers of CF were recruited from one children’s and one adult NHS specialist CF centre: the Cystic Fibrosis Unit at Great Ormond Street Hospital (GOSH) and the West Midlands Adult Cystic Fibrosis Centre at Birmingham Heartlands Hospital. This was a convenient sample drawn from English speaking patients and carriers of CF aged 18 or over who were attending the specialist CF centre for a clinical appointment. Participants were asked to anonymously complete the questionnaire while waiting to see a clinician or to complete it at home and return it via reply-paid post. Details of those given a reply-paid envelope were not recorded, so follow-up was not possible. Health professionals were recruited via an invitation to participate sent by e-mail with a link to the online questionnaire to the member lists of the Association of Genetic Nurse Counsellors (AGNC) (300 recipients) and the Clinical Genetics Society (CGS) (430 recipients).

### 2.3. Questionnaire Design

The questionnaire comprised three sections: (1) DCE choice sets; (2) structured questions about prenatal testing and NIPD; and (3) demographic questions. Attributes for the DCE component of the questionnaire were selected following a series of focus groups with carriers of single gene disorders, where one of the topics raised for discussion was the perceived importance of particular attributes of prenatal tests [19]. The three attributes people felt to be most important were safety, accuracy and time in pregnancy when the test result is received. These attributes form the basis of the DCE. The levels selected for safety (a small risk of miscarriage or no risk of miscarriage), accuracy (90%, 95%, 98% or 100%) and time of test results (8 weeks, 10 weeks, 12 weeks or 14 weeks) represent clinically feasible ranges. The DCE design follows the approach of Street and Burgess [23]. Two attributes had four levels and one attribute had two levels (Supplementary Material, Figure S1A). The number of possible combinations of attributes and levels was statistically reduced from 32 (4^2^ × 2^1^) to eight scenarios using an orthogonal fractional main effects design [24], and a shift of one level was to create eight additional scenarios. The two sets of scenarios were then randomly paired to form the eight choice sets. All levels of each attribute occur with equal frequency (level balance), and within each individual choice set, there is no overlap in attribute levels (minimal overlap). One of the choice sets had a clearly superior test as an option, and this was used as an internal consistency check. The questionnaires for adult patients with CF and carriers of CF asked which test they would prefer to have, and the questionnaires for health professionals asked which test they would prefer to offer. Participants were asked to choose between Test A, Test B or neither (Supplementary Material, Figure S1B). Inclusion of the neither option makes the choice more realistic.

The structured questions around prenatal tests included ranking five attributes of prenatal tests (early testing, accuracy, financial cost, safety and comprehensive information) in order of importance and views on prenatal testing for CF, including views on the pressure to have prenatal testing for CF and who should be offered NIPD for CF. In addition, the service users were asked whether they have had or would have an invasive test for CF and if they would have NIPD if it became available. Demographic questions for service users included age, gender, ethnicity, education and number of children. Demographic questions for health professionals included job title, years in role, age and gender. For the questionnaire, NIPD was described as follows: “NIPD is a non-invasive test that will allow us to use a normal blood sample from the mother’s arm to determine whether or not the baby has CF. Because it is a blood test, there is no risk of miscarriage. Non-invasive tests to identify CF are being developed and are not currently available”. The questionnaires took approximately 20 min to complete. Questionnaires were piloted with 20 carriers of CF to determine whether they could be readily understood, and participants were asked if there were any other important attributes of prenatal tests that were not covered in the questionnaire. No changes were made following the pilot.

### 2.4. Analysis

The DCE preference data were analysed using a conditional logit regression model [25] that included a constant term to reflect the “neither” option [26]. For data entry, the levels for accuracy and time of results were mean centred, and the risk of miscarriage was effect coded. The sign (+ or −) of the coefficients generated in the regression analysis indicates the direction of the preference for each attribute. We anticipated positive coefficients for accuracy and no miscarriage risk, as we expected participants to prefer tests with greater accuracy and safer tests. We anticipated a negative coefficient for the timing attribute, as we expected participants to prefer an earlier test. The preferences of adult patients with CF and carriers of CF were compared to each other and to those of health professionals. In addition, we determined which participants considered multiple attributes when choosing between tests (“traders”) and those who made choices on the basis of one attribute only (“non-traders”). To explore the trade-offs that participants were willing to make between test attributes, we calculated the marginal rates of substitution (MRS) as the ratio of the coefficients of two attributes. The MRS allows direct assessment of how much of one attribute participants are willing to trade for one unit of another attribute and enables a comparison of different attributes on a common scale [21].

Other analyses involved descriptive statistics on single items. On the agreement scale, “Strongly agree” and “Agree”, as well as “Strongly disagree” and “Disagree” were collapsed together to yield a binary index of agreement. The relationship between variables was examined using a chi-square test. For all tests, *p*-values < 0.05 were considered statistically significant. The software package Stata 10.0 [27] (StataCorp., College Station, Texas, TX, USA) was used to perform all analyses.

## 3. Results

### 3.1. Participants

The response rate for patients recruited through the adult CF centre was 91.59% (98/107) and 83.64% (46/55) through the children’s CF centre. The response rate for health professionals was 7.3% (22/300) through the AGNC mailing list and 11.2% (48/430) through the CGS mailing list. Questionnaires were excluded if the consistency question was not answered as expected (patients *n* = 1; health professionals *n* = 0) or if the respondents did not complete the choice set (patients *n* = 1; health professionals *n* = 0). Consequently, a total of 142 service user and 70 health professional questionnaires were included in the analysis. Demographic information for health professionals and service users is summarised in the Supplementary Material, Tables S1 and S2, respectively.

### 3.2. Regression Results

Both service users and health professionals prefer a test with greater accuracy, early testing and no risk of miscarriage (Table 1). These results meet the *a priori* expectations and, thus, support the theoretical validity of the models. All coefficients were statistically significant for both groups. The comparison of service user and health professional regression results shows a statistically significant difference between the coefficients for all three attributes (Table 1). The comparison of people affected with CF and people who are carriers of CF indicated that people affected by CF placed a greater emphasis on safety, but there was no significant difference for accuracy or timing (Table 2). For those affected with CF, the comparison based on gender found that males placed a greater emphasis on safety, but there was no significant difference for accuracy and timing (Table 2). There were not sufficient males who were carriers of CF to allow for a comparison of men and women for the carriers of CF. The participants’ willingness to trade between the attributes was considered (Supplementary Material, Table S3). Notably, 48.9% of people affected with CF and 40.0% of carriers of CF chose tests based solely on there being no miscarriage risk.

**Table 1:** Conditional logit analysis regression results for service users and health professionals.

Attributes	Service users (*n* = 142)		Health professionals (*n =* 70)		Difference
	Coefficient (95% CI) ^a^	*p*-Value	Coefficient (95% CI) ^b^	*p*-Value	*p*-Value
Accuracy	0.160 (0.126 to 0.194)	<0.0001	0.378 (0.318 to 0.438)	<0.0001	<0.0001
Time of results	−0.108 (−0.149 to −0.067)	<0.0001	−0.233 (−0.299 to −0.166)	<0.0001	0.0016
No miscarriage risk	1.960 (1.751 to 2.170)	<0.0001	0.938 (0.639 to 1.238)	<0.0001	<0.0001

CI, confidence interval; ^a^ The number of observations = 3408; pseudo-*R*^2^ = 0.4966; ^b^ The number of observations = 1695; pseudo-*R*^2^ = 0.4746.

**Table 2:** Conditional logit analysis regression results to compare service user subgroups. CF, cystic fibrosis.

Attribute	Service user groups			Affected with CF: gender		
	Affected with CF ^a^ (*n* = 92)	Carrier of CF ^b^ (*n* = 50)	Difference (*p*-Value)	Females ^c^ (*n* = 42)	Males ^d^ (*n* = 50)	Difference (*p*-Value)
	Coefficient (95% CI)	Coefficient (95% CI)		Coefficient (95% CI)	Coefficient (95% CI)	
Accuracy	0.154 (0.111 to 0.197)	0.169 (0.113 to 0.224)	0.6879	0.136 (0.0825 to 0.190)	0.182 (0.119 to 0.245)	0.2751
Time of results	−0.113 (−0.167 to −0.060)	−0.102 (−0.166 to 0.037) *	0.7890	−0.094 (−0.167 to −0.021) *	−0.137 (−0.218 to −0.056) *	0.4390
No miscarriage risk	2.149 (1.873 to 2.425)	1.698 (1.373 to 2.024)	0.0355	1.849 (1.507 to 2.191)	2.512 (2.099 to 2.926)	0.0154

CI, confidence interval; * Coefficient significant at <0.05; All other coefficients significant at <0.0001; ^a^ The number of observations = 2208; pseudo-*R*^2^ = 0.4961; ^b^ The number of observations = 1224; pseudo-*R*^2^ = 0.5221; ^c^ The number of observations = 1032; pseudo-*R*^2^ = 0.4234; ^d^ The number of observations = 1176; pseudo-*R*^2^ = 0.5692.

### 3.3. Marginal Rates of Substitution

Calculation of the MRS confirmed the service user’s strong preference for a test with no risk of miscarriage. Service users were prepared to wait longer and accept lower accuracy compared to health professionals for a test that had no risk of miscarriage (Table 3).

**Table 3:** Marginal rates of substitution.

Attribute	Number of weeks that respondents are prepared to wait		Reduction in accuracy (%) that respondents are prepared to accept	
	Service users	Health professionals	Service users	Health professionals
Test with no risk of miscarriage	18.15 (1.960/−0.108)	4.03 (0.938/−0.233)	12.25 (1.960/0.160)	2.48 (0.938/0.378)
Test with 5% greater accuracy	7.41 (0.160/−0.108 × 5)	8.11 (0.378/−0.233 × 5)	-	-
Early test	-	-	0.68 (−0.108/0.160)	0.62 (−0.233/0.378)

### 3.4. Ranking of Attributes

Participants were asked to rank five test attributes in order of importance: early testing, accuracy, financial cost, safety and comprehensive information (Supplementary Material, Tables S4 and S5). Rankings supported the regression results, with 62% of service users ranking safety highest, whereas 81% of health professionals ranked accuracy as the most important attribute. Cost was ranked lowest by both groups.

### 3.5. Views on Prenatal Testing and the Introduction of NIPD

Service users were asked about their willingness to have prenatal testing for CF (Table 4). More than half of the participants (56.5%) said they would not have an invasive diagnostic test for CF. Amongst those that said they would have an invasive test, the most common reason given was “to plan and prepare for the possible birth of a baby with CF” (62.3%), with a smaller proportion (32.1%) having testing to “help make a decision about whether or not to continue the pregnancy”. Over half of the participants said they would never have an invasive test because of the risk of miscarriage. When asked whether they would have NIPD for CF if it was available, 94.9% of participants said they would and 90% said that they would be prepared to pay for the test. Around 10% of respondents would not be prepared to pay for NIPD. There was no significant difference between the responses of those affected with CF and those who were carriers of CF.

**Table 4:** Service user uptake of prenatal testing. NIPD, non-invasive prenatal diagnosis.

Questions regarding uptake of prenatal testing	Total (*n* = 142)	Affected with CF (*n* = 92)	Carrier of CF (*n* = 50)	*p-*Value
**Have had/likely to have an invasive test for CF**				0.996
Strongly Agree/Agree	57 (43.5%)	37 (43.5%)	20 (43.5%)	
Strongly Disagree/Disagree	74 (56.4%)	48 (56.5%)	26 (56.5%)	
**Reason for choosing to have a diagnostic test**				0.288
To plan and prepare for the possible birth of a baby with CF	33 (62.3%)	22 (66.7%)	11 (55.0%)	
To help make a decision about whether or not to continue the pregnancy	17 (32.1%)	9 (27.3%)	8 (40.0%)	
**Reason for choosing to have a diagnostic test**				0.288
Because my family or my partner would want me to	2 (3.8%)	2 (6.0%)	0 (0%)	
Because it is offered as part of the antenatal service	1 (1.9%)	0 (0%)	1 (5.0%)	
Other	0 (0%)	0 (0%)	0 (0%)	
**Would never have an invasive test because would not consider termination of pregnancy**				0.447
Strongly Agree/Agree	63 (47.0%)	43 (49.4%)	20 (42.5%)	
Strongly Disagree/Disagree	71 (53.0%)	44 (50.5%)	27 (57.4%)	
**Would never have an invasive test because of the risk of miscarriage**				0.466
Strongly Agree/Agree	71 (56.4%)	52 (58.4%)	19 (51.3%)	
Strongly Disagree/Disagree	55 (43.7%)	37 (41.6%)	18 (48.6%)	
**Would have NIPD if available**				0.252
Strongly Agree/Agree	130 (94.9%)	84 (93.3%)	46 (97.9%)	
Strongly Disagree/Disagree	7 (5.1%)	6 (6.7%)	1 (2.1%)	
**Willingness to pay for NIPD**				0.126
≤£50	56 (41.2%)	32 (36.0%)	24 (51.1%)	
£100–200	53 (39.0%)	40 (44.9%)	13 (27.7%)	
≥£200	14 (10.3%)	6 (6.7%)	8 (17.0%)	
Not prepared to pay	13 (9.6%)	11 (12.4%)	2 (4.3%)	

Service users and health professionals were asked their views on the pressure to have prenatal testing for CF and whether the introduction of NIPD would increase this pressure (Table 5). The majority of both service users (60.6%) and health professionals (74.6%) thought that there was no pressure on couples at risk of having a child with CF to have prenatal testing. Service users who felt there was pressure to have testing reported that the three main sources of this pressure were a partner, family members and health professionals. Approximately half of the service users (47.0%) and half of the health professionals (55.2%) thought that the pressure to have prenatal testing would increase if NIPD for CF became available. There were no significant differences between the responses of health professionals, people affected with CF or people who are carriers of CF.

**Table 5:** Views on the pressure to have prenatal testing.

Questions regarding pressure to have prenatal testing	Total service users (*n* = 142)	Affected with CF (*n* = 92)	Carriers of CF (*n* = 50)	Health professionals (*n* = 70)	*p*-Value
**There is pressure on women at risk of having a child with CF to have a diagnostic test in pregnancy**					0.075
Strongly Agree/Agree	54 (39.4%)	38 (43.2%)	16 (32.7%)	17 (25.8%)	
Strongly Disagree/Disagree	83 (60.6%)	50 (56.8%)	33 (67.3%)	49 (74.2%)	
If you agree, where do you think this pressure comes from *					
Partner	27 (26.7%)	21 (27.6%)	6 (24.0%)	15 (32.6%)	
Family members	29 (28.7%)	22 (28.9%)	7 (28.0%)	13 (28.3%)	
Health professionals	24 (23.8%)	20 (26.3%)	4 (16.0%)	5 (10.9%)	
Society in general	14 (13.9%)	8 (10.5%)	6 (24.0%)	6 (13.0%)	
Your cultural or religious community	1 (1.0%)	0 (0%)	1 (4.0%)	7 (15.2%)	
Other	6 (5.9%)	5 (6.6%)	1 (4.0%)	0 (0%)	
**The availability of NIPD will increase the pressure to have prenatal testing**					0.088
Strongly Agree/Agree	52 (42.6%)	40 (46.5%)	12 (33.3%)	37 (56.1%)	
Strongly Disagree/Disagree	70 (57.4%)	46 (53.5%)	24 (66.7%)	29 (43.9%)	

* Participants could choose up to three responses.

**Figure 1:**
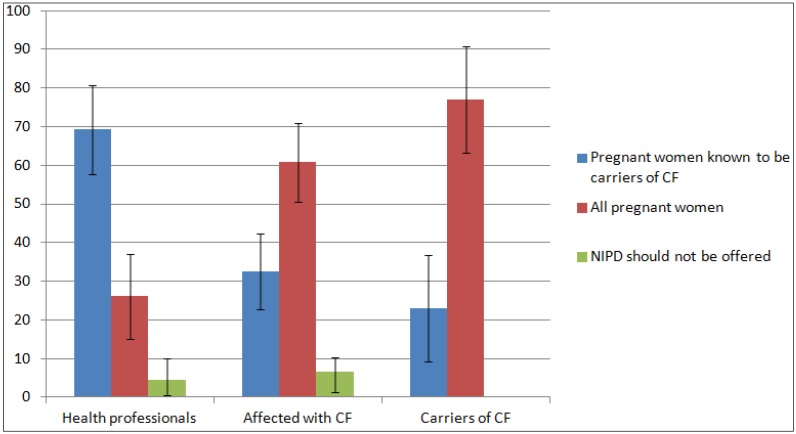
Who should be offered NIPD for CF?

Service users and health professionals were asked who they thought should be offered NIPD for CF (Figure 1). The majority of health professionals (68.2%) thought that NIPD should only be offered to pregnant women who are known to be carriers of CF, whereas the majority of participants who are affected with CF (60.1%) or carriers of CF (76.9%) thought NIPD for CF should be offered to all pregnant women.

## 4. Discussion

In this study, we have examined the preferences of potential service users and health professionals for prenatal tests for CF. A DCE was designed to explore the relative importance placed on key attributes of hypothetical diagnostic tests for CF, taking into account the key clinical features of NIPD relative to invasive tests. While both service users and health professionals preferred safe tests with high accuracy that were conducted early in pregnancy, differences between groups arose in the emphasis placed on each of these attributes. Service users were prepared to wait longer and accept lower accuracy than health professionals if the test had no risk of miscarriage. The importance of safe testing for service users was further highlighted, as approximately half of this group did not trade on the test choices and chose only those tests that had no risk of miscarriage. These findings are analogous to those of a DCE study looking at preferences for attributes of invasive and non-invasive tests for Down’s syndrome, wherein accuracy was the key driver of decisions for health professionals and safety was most important for pregnant women [18]. Health professionals offering NIPD for CF in the future need to be aware that their views about the importance of test attributes may differ from the views of their clients. As safety is such a clear priority when service users are making decisions about whether to have prenatal testing, care must be taken to ensure that the other attributes of NIPD, including the disadvantages, are presented.

More than half of the service users indicated that they would not have invasive testing for CF and that the risk of miscarriage was a barrier to testing. In addition, our findings, like those of other studies [5,28,29,30,31,32], indicate that while many couples who would have invasive testing for CF make this choice to guide decisions about termination of pregnancy, a large proportion take up testing so that they can plan and prepare for the birth of a baby affected with CF and would not consider termination. Accordingly, over 90% of participants said they would have NIPD for CF if it was offered. Overall, these results suggest that uptake of NIPD is likely to be high and will include many couples who would currently decline invasive testing due to the risk of miscarriage and a large proportion who would want testing for information only. This supports the findings of qualitative studies, which also suggested that uptake of prenatal testing for single gene disorders would increase if NIPD became available [19,20]. The high predicted uptake for NIPD does, however, need to be interpreted with caution, since predicted uptake may differ from actual uptake, as has been reported in other studies of genetic testing [33,34,35]. As such, research to monitor actual uptake when NIPD for CF is implemented will be important.

Other studies have shown that reproductive choices around prenatal diagnosis for CF and termination of pregnancy change over time, and what people said they would do was often different from their final decisions [29]. This notion of reproductive choices being dynamic is particularly important to note if a large proportion of couples who would not previously have had prenatal testing take up NIPD, as many may find themselves making a decision about the termination of pregnancy, which they would not have been confronted with in the past. For these reasons, it will be important to take time in pre-test counselling to talk through the implications of the test and the possibility that people may change their mind about the termination of pregnancy when the test result is given. It is also critical that individualised support is available through post-test counselling.

One of the concerns most often raised about NIPD is that people may feel pressured to have the test merely because it is easy to do and has no risk of miscarriage [36,37,38]. Indeed, previous research around NIPD for the single gene disorders, CF, sickle cell disease and thalassaemia, highlighted concerns from both potential service users and health professionals that the perceived ease of a blood test may bring increased pressure to have diagnostic testing during pregnancy [19,20]. In the current study, this concern was again voiced, with the majority of health professionals (55.2%) and 43.5% of service users feeling that the availability of NIPD would increase the pressure to have a prenatal test for CF. In addition, the DCE results indicate that the option of a test with no risk of miscarriage is highly influential. Health professionals offering NIPD for CF need to be conscious of the potential for NIPD to engender feelings of pressure and take care when counselling to ensure that this is not the motivation for prenatal testing. Being aware and willing to adjust approaches to counselling is particularly important, as service users cited health professionals as one of the three main sources of pressure to have prenatal testing. In particular, it is important not to “sell” NIPD by virtue of its safety, so that couples can make prenatal testing choices in line with their personal beliefs and values.

There was a significant difference between service user and provider views on who should be offered NIPD for CF, with service users suggesting NIPD should be offered to all pregnant women and health professionals feeling this test should be reserved for known carriers. Further research is needed to understand the nuances of this difference. It is possible that health professionals may be considering wider issues, such as cost to the NHS. Moreover, service users may be responding to the fact that there is no carrier screening programme for CF in the UK and carrier status is often not picked up until after the birth of a baby with CF, as 95% of carriers have no family history of the condition [39]. Prenatal carrier screening for CF is recommended for all pregnant women in some countries, including the U.S. [40] and Australia [41], but is not routinely offered in the UK. The UK’s National Screening Committee is currently reviewing carrier screening for CF in pregnancy [42]. Previous research conducted in the UK has shown that people affected with CF and parents of children with CF support population carrier screening during pregnancy [43]. The cost of NIPD would be a key factor in determining whether the test could be offered to all pregnant women. In addition, it is not yet clear how many CF mutations it will be possible to test for with NIPD and whether this will match current carrier screening panels that test for 23 different CF mutations.

### Limitations

A number of issues may limit the generalizability of our findings. Most notably, the response rate for health professional recruitment was low (9.7%). However, this response rate is equivalent to similar studies recruiting health professionals by e-mail invitation to member lists of professional bodies [44]. The low response rate may mean there is a sampling bias towards those with pre-existing interests or particular concerns about NIPD, and as such, the responses may not represent the wider views of genetic specialists. The response rate for potential service user recruitment was high for both people affected with CF and people who are carriers of CF. However, while approximately half of the participants who were affected with CF were men, only a small number of men who are carriers of CF were recruited. This may impact findings, as there is evidence to suggest that men and women may have conflicting views and can take on different roles in reproductive decision making about CF [5,32]. Gender differences were in fact seen in this study, as men affected with CF were found to place a greater emphasis on test safety than women affected with CF. Infertility and the need for assisted reproductive technology for men who are affected with CF are likely to be major factors in their additional emphasis on choosing safe prenatal tests; however, further research to explore the motivations is needed. It would also be valuable to see if this gender-based difference is present in carriers of CF. In addition, as our recruitment focussed on adult and paediatric CF clinics, we are missing the views of parents of children with CF in the case where the child has died. In addition, only a small number of carriers of CF who do not have children were recruited, and the views of this group may differ to those of carriers with children who may have more direct experience of living with CF. As with any stated preference study, the choices made by participants do not necessarily reflect the choices that would be made in real life. The DCE only included three attributes of prenatal tests, when real-life decisions must incorporate many more considerations. In addition, the DCE design does not explore the reasoning behind the choices made and give insight into how the tests were perceived. Finally, as discussed earlier, participants’ stated uptake of NIPD is hypothetical and may not necessarily correlate with actual uptake.

## 5. Conclusions

Successful implementation of NIPD for CF and other single gene disorders must consider the views and preferences of a range of stakeholders. In this study, we found that potential service users and health professionals place different values on the test attributes of safety, accuracy and timing when making decisions about prenatal testing for CF. Implementation of NIPD for CF into clinical practice must consider these differences to ensure that the needs of all stakeholders are met. It was clear that when making decisions about testing, service users placed great emphasis on test safety. While it is likely that as a safe test, NIPD will be welcomed by couples and uptake will be high, pre-test counselling must include issues beyond safety to address the concerns raised regarding the availability of NIPD increasing the pressure to undergo prenatal testing. Even for service user groups who are clearly very familiar with the condition, pre-test counselling and informed consent processes that allow time for reflection are needed to make certain that attributes of the test beyond safety are discussed and that the implications of the result are considered. The importance of thorough pre- and post-test counselling emphasises the need to maintain specialist care pathways for the delivery of NIPD, so testing is offered by health professionals specifically trained in counselling for prenatal testing. Future research at the time of clinical implementation will be required to guide continued service delivery, as predicted uptake may not reflect what actually happens in practice. In addition, discussion and debate is needed as to how NIPD is offered, as service users are clearly in favour of testing being available to all pregnant women, regardless of whether the carrier status of CF is known.

## Supplementary Files

Supplementary File 1
