# Supplementary material for: Preferences for Prenatal Tests for Cystic Fibrosis: A Discrete Choice Experiment to Compare the Views of Adult Patients, Carriers of Cystic Fibrosis and Health Professionals
Source: J Clin Med. 2014 Feb 14;3(1):176–90. doi: 10.3390/jcm3010176 (PMC4449661; doi:10.3390/jcm3010176)
Supplement: Supplementary File 1 [file jcm-03-00176-s001.pdf]

Supplementary Information

Figure S1. Discrete choice experiment design. (**A**) Attributes and levels used in the discrete choice experiment; (**B**) Example of a discrete choice experiment choice set.

Attribute	Levels
Accuracy	90%, 95%, 98%, 100%
Time of results (gestation in weeks)	8, 10, 12, 14
Risk of miscarriage	Small risk (1%), No risk

A

Choice 1	Test A	Test B
Accuracy	95%	100%
Time of results	10 weeks	12 weeks
Risk of miscarriage	Small risk (1%)	No risk

Which test would you prefer (*tick one box only*)?

Test A ☐ Test B ☐ Neither ☐

B

Table S1. Health professional demographic data.

Demographic data	Total (*n* = 70)
**Age in years**	
Mean (SD)	43.3 (10.98)
**Gender**	
Female	50 (86.2%)
Male	8 (13.8%)
**Profession**	
Clinical geneticist	48 (68.6%)
Genetic counsellor	22 (31.4%)
**Years in profession**	
≤5	9 (15.0%)
6–15	25 (43.3%)
16–25	17 (28.3%)
≥26	9 (15.0%)
**Current practice location**	
England (London)	9 (18.8%)
England (Regional)	24 (50.0%)
Scotland	5 (10.4%)
Northern Ireland	1 (2.1%)
Outside the UK	7 (14.6%)
Not in current practice	2 (4.2%)

In some cases, numbers may not add up to the total *n* due to missing data. Percentages may not add up to 100 due to rounding.

Table S2. Service user demographic data.

Demographic data	Total (n = 142)	Affected with CF (n = 92)	Carrier of CF (n = 50)
**Gender**			
Female	78 (54.9)	42 (45.7%)	36 (72.0%)
Male	64 (45.1)	50 (54.4%)	14 (28.0%)
**Age in years**			
Mean (SD)	32.19 (9.84)	29.33 (9.85)	37.46 (7.4)
**Ethnicity**			
White	140 (99.3%)	91 (100%)	49 (98.0%)
Other	1 (0.71%)	0 (0%)	1 (2.0%)
**Highest qualification**			
No qualification	1 (0.7%)	0 (0%)	1 (2.0%)
High school	48 (34.8%)	34 (38.6%)	14 (28.0%)
Some college or other training	59 (42.8%)	37 (42.1%)	22 (44.0%)
Degree or equivalent	30 (21.7%)	17 (19.3%)	13 (26.0%)
**Relationship status**			
Married/In a relationship	103 (73.6%)	65 (71.4%)	38 (77.6%)
Separated/Divorced	6 (4.3%)	1 (1.1%)	5 (10.2%)
Widowed	1 (0.7%)	0 (0%)	1 (2.0%)
Single	30 (21.4%)	25 (27.5%)	5 (10.2%)
**Religious faith**			
Yes	44 (31.9%)	23 (25.3%)	21 (44.7%)
No	94 (68.1%)	68 (74.7%)	26 (55.3%)
**Currently pregnant**			
Yes	4 (2.8%)	1 (1.1%)	3 (6.0%)
No	138 (2.8%)	91 (98.9%)	47 (94.0%)
**Number of children**			
None	79 (56.4%)	75 (83.3%)	4 (8.0%)
1	33 (23.6%)	11 (12.2%)	22 (44.0%)
2 or more	28 (20.0%)	4 (4.5%)	24 (48.0%)
**Child with cystic fibrosis?**			
Yes	44 (31.9)	0 (0%)	44 (91.7%)
No	94 (68.1)	90 (100%)	4 (8.3%)

In some cases, numbers may not add up to the total n due to missing data. Percentages may not add up to 100 due to rounding.

Table S3. Service users and health professionals who did not make trades with their choices.

Test Attributes	Affected with CF (n = 92)	Carrier of CF (n = 50)	Health Professionals (n = 70)
Chose tests with highest accuracy for all options	5 (5.4%)	4 (8.0%)	5 (7.1%)
Chose tests with the earliest time for all options	1 (1.1%)	2 (4.0%)	1 (1.4%)
Chose tests with no risk for all options	45 (48.9%)	20 (40.0%)	1 (1.4%)
Chose neither for all options	3 (3.26%)	0 (0%)	1 (1.4%)
Total	54 (58.7%)	26 (52.0%)	8 (11.4%)

Table S4. Ranking of test attributes: Service users.

Attribute	Ranking (%) *				
	1	2	3	4	5
Early test	8.27	12.78	31.58	35.34	12.03
Accuracy	18.80	48.87	23.31	9.02	0
Cost	0	2.26	4.51	10.53	82.71
Safety	62.41	18.80	11.28	7.52	0
Full information	12.03	17.29	28.57	36.84	5.26

* n = 133.

Table S5. Ranking of test attributes: Health professionals.

Attribute	Ranking (%) *				
	1	2	3	4	5
Early test	4.69	28.13	50.00	14.06	3.13
Accuracy	81.25	15.63	3.13	0	0
Cost	1.56	0	9.38	39.06	50.00
Safety	14.06	50.00	29.69	6.25	0
Full information	0	4.69	9.38	39.06	46.88

* n = 64.
